# The GH51 α-l-arabinofuranosidase from *Paenibacillus* sp. THS1 is multifunctional, hydrolyzing main-chain and side-chain glycosidic bonds in heteroxylans

**DOI:** 10.1186/s13068-016-0550-x

**Published:** 2016-07-08

**Authors:** Hanen Bouraoui, Marie-Laure Desrousseaux, Eleni Ioannou, Pablo Alvira, Mohamed Manaï, Caroline Rémond, Claire Dumon, Narcis Fernandez-Fuentes, Michael J. O’Donohue

**Affiliations:** UBMB, Université de Tunis El Manar, BP 94, 1068 Rommana, Tunisia; CNRS, INRA, INSA, LISBP, Université de Toulouse, Toulouse, France; INRA, FARE, Université de Reims Champagne Ardenne, 2, Esplanade Roland Garros, 51100 Reims, France; Institute of Biological, Environmental and Rural Sciences, Aberystwyth University, Aberystwyth, SY23 3DA Ceredigion UK; Laboratoire des Ressources Sylvo-Pastorales, Institut Sylvo-Pastoral de Tabarka, Institution de la Recherche et de l’Enseignement Supérieur Agricoles, Université de Jendouba, Jendouba, Tunisia

**Keywords:** Glycoside hydrolase, Xylanase, Wheat bran, Enzyme cocktails, Biomass

## Abstract

**Background:**

Conceptually, multi-functional enzymes are attractive because in the case of complex polymer hydrolysis having two or more activities defined by a single enzyme offers the possibility of synergy and reduced enzyme cocktail complexity. Nevertheless, multi-functional enzymes are quite rare and are generally multi-domain assemblies with each activity being defined by a separate protein module. However, a recent report described a GH51 arabinofuranosidase from *Alicyclobacillus* sp. A4 that displays both α-l-arabinofuranosidase and β-d-xylanase activities, which are defined by a single active site. Following on from this, we describe in detail another multi-functional GH51 arabinofuranosidase and discuss the molecular basis of multifunctionality.

**Results:**

THSAbf is a GH51 α-l-arabinofuranosidase. Characterization revealed that THSAbf is active up to 75 °C, stable at 60 °C and active over a broad pH range (4–7). THSAbf preferentially releases para-nitrophenyl from the l-arabinofuranoside (*k*_cat_/*K*_M_ = 1050 s^−1^ mM^−1^) and to some extent from d-galactofuranoside and d-xyloside. THSAbf is active on 4-O-methylglucuronoxylans from birch and beechwood (10.8 and 14.4 U mg^−1^, respectively) and on sugar beet branched and linear arabinans (1.1 ± 0.24 and 1.8 ± 0.1 U mg^−1^). Further investigation revealed that like the *Alicyclobacillus* sp. A4 α-l-arabinofuranosidase, THSAbf also displays endo-xylanase activity, cleaving β-1,4 bonds in heteroxylans. The optimum pH for THASAbf activity is substrate dependent, but ablation of the catalytic nucleophile caused a general loss of activity, indicating the involvement of a single active center. Combining the α-l-arabinofuranosidase with a GH11 endoxylanase did not procure synergy. The molecular modeling of THSAbf revealed a wide active site cleft and clues to explain multi-functionality.

**Conclusion:**

The discovery of single active site, multifunctional enzymes such as THSAbf opens up exciting avenues for enzyme engineering and the development of new biomass-degrading cocktails that could considerably reduce enzyme production costs.

**Electronic supplementary material:**

The online version of this article (doi:10.1186/s13068-016-0550-x) contains supplementary material, which is available to authorized users.

## Background

Heteroxylans (commonly referred to as xylans) composed of β-1,4-linked d-xylosyl subunits constitute an important class of polysaccharides that are widespread throughout the plant kingdom, notably in flowering plants [1]. In both perennial and annual grassy species, the main chains of xylans are mainly decorated with l-arabinofuranosyl moieties. However, depending on the exact origin of the source material, the frequency of the arabinofuranosyl decorations and the bonds linking them to the xylan main chain are different [2].

One consequence of the structural complexity of heteroxylans is the diversity of the enzymes that are required to break them down into sugars. This is illustrated by the complex enzymatic arsenals deployed by microbial plant pathogens, plant saprophytes and gut microbiota [3]. Generally, the core enzyme activities produced by xylanolytic microorganisms are endo-β-1,4-d-xylanases (EC 3.2.1.8) and β-1,4-d-xylosidases (EC 3.2.1.37), which act on β-1,4 bonds that link d-xylosyl moieties in polysaccharides and oligosaccharides, and α-l-arabinofuranosidases (EC 3.2.1.55) that hydrolyze the 1,2 and 1,3 glycosidic that link α-l-arabinofuranoyl side chains to the main chain, although the actual list of enzyme activities is much longer.

Regarding different α-l-arabinofuranosidases (or Abfs), enzymes displaying this activity are quite widespread in the present CAZy classification system (http://www.cazy.org), although families GH51, 43 and 62 are the main families. Of these, GH51 is by far the largest family, with a majority of its members being of bacterial origin. Structural data obtained for several different members of the family confirm that all enzymes in GH51 possess a catalytic domain that displays (β/α)_8_-barrel architecture, and additionally all bear a C-terminal β-sandwich domain of unknown function [4]. GH51 enzymes operate through a double displacement mechanism that leads to the overall retention of the anomeric configuration of the carbon at the scissile bond [5] and involves two conserved glutamic acid residues [6]. Regarding the finer structural details of GH51 enzymes, work on *Thermobacillus xylanilyticus* (*Tx*Abf, UNIPROT O69262, PDB 2VRK and 2VRQ) has revealed two putative active site configurations. In the so-called open configuration, the β2α2 loop bearing Trp99 is distant from the active site. However, in the closed configuration the same loop closes down onto and forms the active site, with Trp99 contributing to the topology of the −1 subsite that accommodates non-reducing l-arabinosyl moieties.

So far, the majority of characterized GH51 family members have been shown to be α-l-arabinofuranosidases (EC 3.2.1.55), although a few display endoglucanase (EC 3.2.1.4) activity [7]. Previously, Hövel et al. [8] rationalized the co-occurrence of these two activities within GH51 by considering the structural similarities between a GH51 α-l-arabinofuranosidase and a GH5 endoglucanase and specifically showing how d-glucosyl moieties can conceivably be accommodated in the −1 and +1 subsites of the GH51 enzyme. In addition to these two substrate specificities, studies have revealed that GH51 Abfs can often hydrolyze the glycosidic bond in aryl-glycosides, such as *para*-nitrophenyl-β-d-xylopyranoside or (*p*NP-Xyl*p*) or *para*-nitrophenyl-β-d-galactofuranoside (*p*NP-Gal*f*), the explanation for this flexibility being found in the stereochemical relatedness of α-l-arabinosyl, β-d-xylopyranosyl and β-d-galactofuranosyl moieties [8, 9]. Nevertheless, apart from rare examples [10], these activities are often minor and are not evidenced when GH51 Abfs are used to hydrolyze heteroxylan or xylooligosaccharides [11], unlike the clearly bifunctional α-l-arabinofuranosidases/β-d-xylopyranosidases belonging to other GH families, such as GH3 and 43 [12, 13].

Conceptually, bifunctional enzymes are attractive, because in the case of complex polymer hydrolysis, the two activities are likely to be complementary and catalyze consecutive reactions [14]. In the context of heteroxylan hydrolysis, it is easy to imagine that bifunctional systems of the type α-l-arabinofuranosidase/β-d-xylopyranosidases or α-l-arabinofuranosidase/β-d-xylanase would be particularly useful and, indeed, the literature abounds with examples of the former type. However, only two examples of naturally occurring α-l-arabinofuranosidase/β-d-xylanases have been reported. The Xln23 from *Streptomyces chattanoogensis* is bimodular, containing two catalytic domains; thus the activities are displayed by two distinct active sites [15]. Similarly, artificial enzymes, such as the chimeras designed by Fan et al. [16, 17] are also bimodular. On the other hand, a recently reported GH51 arabinofuranosidase from *Alicyclobacillus* sp. A4 appears to possess a single catalytic domain that is responsible for both α-l-arabinofuranosidase and β-d-xylanase activities, although no xylosidase activity was evidenced [18]. This novel finding is intriguing, because it is not easy to understand how the active site topology of typical GH51 arabinofuranosidases can accommodate heteroxylan in a productive manner leading to the hydrolysis of β-(1-4) bonds linking main-chain d-xylosyl moieties.

From a technological standpoint, Abfs, β-d-xylopyranosidases and β-d-xylanases are useful for a wide variety of commercial applications, including the pre-hydrolysis of non-digestible fibers in animal feed and the production of sugar syrups from lignocellulosic biomass, being particularly vital when targeting xylan-rich agricultural co-products, such as brans from corn and wheat, and hardwoods. Therefore, it is easy to appreciate that enzymes displaying several xylanolytic activities could be useful to reduce the complexity and the cost of enzyme cocktails [19] or, alternatively, to limit the number of coding sequences needed to construct designer xylanolytic microorganisms [20].

Consistent with the recent report by Yang et al. [18], we describe herein another multifunctional GH51 arabinofuranosidase that is produced by a bacterium isolated from a Tunisian hot spring. The detailed study of this apparently trifunctional enzyme has provided us with some clues as to how all three substrate specificities can be defined by a single catalytic site.

## Methods

### General chemicals and reagents

Most chemicals and reagents used in enzyme assays were purchased from Sigma-Aldrich Chimie S.a.r.l. (Lyon, France) unless stated otherwise. However, *p*NP-Gal*f* was prepared in-house using a published protocol [9]. Polysaccharides and XOS for calibrating HPAEC experiments were purchased from Megazyme International (Wicklow, Ireland), although the monosaccharides l-arabinose, d-xylose and oat spelt xylan were purchased from Sigma-Aldrich. Destarched wheat bran was a gift from ARD (Pomacle, France).

### Genomic DNA preparation

A moderately thermophilic strain was isolated from a soil sample collected in south Tunisia (Gafsa region) using a batchwise enrichment procedure. Briefly, bacteria were grown in aerobic conditions at 55 °C with shaking in a previously described XE medium [21, 22], which contains 0.3 g/L KH_2_PO_4_, 0.6 g/L NaCl, 12 g/L MgSO_4_·7H_2_O, 0.08 g/L CaCl_2_·2H_2_O, 1 g/L NH_4_Cl, 2 g/L yeast extract), containing 1 % v/v vitamin and 1 % v/v minerals and 5 g/L oat spelt xylan as the sole carbon source. Genomic DNA (gDNA) was isolated from a fresh culture of this strain, essentially using the method described by Harwood and Cutting [23]. After thorough extraction using an aqueous phenol/chloroform/isoamyl alcohol (50:48:2 v/v) solution saturated with 10 mM Tris, 1 mM EDTA, pH 8.0, the gDNA was precipitated using isopropanol and recovered using a glass Pasteur pipette. While attached to the pipette, the gDNA was washed using 70 % (v/v) ethanol and then dissolved in 10 mM Tris, 1 mM EDTA, pH 8.0.

## 16s rDNA cloning and sequencing

Using the gDNA as template and two universal 16s rDNA primers, E8F and E154R (see below), the 16s rDNA of the bacterial isolate was amplified by PCR, employing the high-fidelity Pfu Turbo DNA polymerase (Promega, WI, USA). The amplicon was purified using the QIAquick PCR purification kit (Qiagen S.A., Courtaboeuf, France), ligated to the linear, blunt-ended vector pST1Blue (Novagen, Merck, Darmstadt, Germany) and the reaction mixture was used to transform *E. coli* XL1Blue. After isolation of positive clones, the recombinant plasmid containing the 16s rDNA fragment was amplified and prepared as plasmid DNA using standard methods. This DNA was submitted for sequencing, which was performed by MWG Biotech (Germany). Finally, the sequence data (GENBANK accession number AM283040) was compared to all of the type strain accessions in the ribosomal database (http://www.rdp.cme.msu.edu/). Oligonucleotide primers used to isolate the 16s rDNA of the bacterial isolate were:

E8F 5′ AGAGTTTGATCCTGGCTCAG 3′,

E1541R 5′ AAGGAGGTGATCCANCCRCA 3′.

### Isolation and cloning of the gene encoding THSAbf

Genomic DNA was digested in individual reactions using four different restriction endonucleases (*Hae*III, *Rsa*I, *Alu*I et *Dpn*I) that generate blunt DNA fragments, according to standard molecular biology protocols [24]. Following digestion, DNA fragments were size separated by electrophoresis on an agarose gel, employing low melting point agarose (0.8 % w/v). DNA fragments in the size range 2–7 kb were recovered by excising the relevant zones of the gel and fragments were purified using the Qiaquick gel extraction kit (Qiagen, Courtaboeuf, France), before being ligated to the *Eco*RV-linearized vector pSMART-LCKan. After an appropriate incubation period, aliquots of the ligation mixture were used to transform E. cloni® 10G *Escherichia coli* chemically competent cells (Lucigen Corp, Middleton, WI, USA), which were subsequently cultured on solid LB agar medium containing kanamycin (30 µg mL^−1^) and 5-bromo-4-chloro-3-indolyl-β-d-xylopyranoside (X-Xyl, Glycosynth Ltd, Cheshire, UK). The latter (40 µL aliquot of a solution of 20 mg/mL in DMSO) was applied to the surface of the solid medium to enable the selection of clones expressing β-d-xylopyranosidase activity. The sequence of one positive clone encoding the family GH51 Abf, designated THSAbf, was amplified by PCR using the primers below and inserted between the *Nde*1 and *Eco*R1 sites of the expression vector pET21a (+), thus creating the plasmid pET21-THSAbf that was used to transform the *E. coli* strain JM109 DE3. This plasmid encodes a recombinant version of THSAbf bearing a C-terminal (His)_6_ tag.

FwAbf (*Nde*1underlined) 5′ GGATCAGCATATGTCATCAACAGCACCGCG 3′,

RevAbf (*Eco*R1 underlined) 5′ CCATGAATTCGCAGCTCTCGCTTGACCCAG 3′.

### Creation of the mutant E296A

To create an inactive variant of THSAbf, site-directed mutagenesis was performed to replace the amino acid E296 (predicted catalytic nucleophile) by an alanine. This was achieved using the QuickChange II site-directed mutagenesis kit (Agilent Technologies, Les Ulis, France) and forward and reverse mutagenic primers:

F-E296A 5′ GTACCTCAGCTTCGACGCGTGGAACGTTTGGTAC 3′,

R-E296A 5′ GTACCAAACGTTCCACGCGTCGAAGCTGAGGTAC 3′.

Mutagenesis was performed using a thermocycler programmed to perform 16 cycles of annealing (95 °C, 30 s and 55 °C, 1 min) and primer elongation at 68 °C for 5 min. Afterward, the mutated plasmid was used to transform *E. coli* strain JM109 DE3. The mutant enzyme E296A was expressed and purified as described below.

### Protein expression

Protein expression was conducted using standard procedures and single colonies were grown at 37 °C in liquid LB medium containing ampicillin (100 µg mL^−1^). A non-standard procedure for pT7-driven expression was used to express THSAbf, since no IPTG was used to induce protein expression. Briefly, a pre-culture established using a single colony *E. coli* strain JM109 DE3 bearing pET21-THSAbf was grown for 16 h at 37 °C with shaking (140 rpm) in LB medium containing ampicillin. This culture was then diluted (1:100 v/v) with fresh LB/ampicillin medium and grown at 37 °C until the OD_600nm_ of the culture reached 0.5 absorbance unit (generally 4 h). At this point, the culture was cooled on ice and then submitted to centrifugation (5000×*g*, 12 min, 4 °C), thus recovering the cells for protein purification.

### Purification of THSAbf

To purify recombinant THSAbf, bacterial cells were suspended in 20 mL of Talon® buffer (Tris–HCl 20 mM, NaCl 300 mM, pH 8) and frozen at −20 °C. After defrosting on ice, the cell suspension was sonicated (0.5 s pulse followed by 0.5 s pause, during a total of 4 min using a ¾-inch probe operating at 30 % amplitude), maintaining the cells on ice throughout. Solid cellular debris was separated from soluble cellular extract by centrifugation (20 min at 15,000×*g*, 4 °C), with the latter being filtered (0.45 µm Minisart syringe filter, Sartorius) before being applied to a gravity flow column containing 3 mL of Talon® metal affinity resin (Clontech, La Jolla, CA, USA). The column was washed successively with 20 mL of Talon® buffer, 7.5 mL of Talon® containing 5 mM imidazole, pH 8, and 7.5 mL of Talon® containing 10 mM imidazole, pH 8. Finally, THSAbf was eluted in 15 mL (applied in 7.5 mL aliquots) of Talon® containing 100 mM imidazole, pH 8, and the collected fractions were dialyzed against 250 volumes of 50 mM sodium phosphate (2 successive baths), pH 7, before being filtered using a sterile Minisart Sartorius filter (0.22 µm) and stored at 4 °C. The final concentration of the THSAbf solutions was determined by measuring the absorbance at 280 nm and applying the Lambert–Beer relationship, using theoretical values for Mw (57,223 Da) and molar extinction coefficient of *ɛ* = 112,230 M^−1^ cm^−1^.

### Bioinformatics and structural modeling of THSAbf–ligand complexes

Routine DNA sequence analysis was performed using Bioedit 7.2.5, while the analysis of 16s rRNA was performed using SILVA, the rRNA database project (http://www.arb-silva.de/). To perform phylogenetic analysis, a set of complete sequences was extracted from family GH51 in the CAZy database (http://www.cazy.org/GH51.html), and then redundant sequences were removed using CD-HIT, fixing the sequence identity cutoff at 1.0. Subsequently, 984 non-redundant sequences were submitted to BLASTCLUST (part of the Bioinformatics Toolkit of the Max Planck Institute for Developmental Biology at http://www.toolkit.tuebingen.mpg.de/blastclust/), using 80 % sequence identity and 70 % sequence coverage as run settings. Sequences representing 577 clusters were then aligned using CLUSTAL OMEGA (http://www.toolkit.tuebingen.mpg.de/clustalw) and the phylogenetic tree was plotted using Figtree 1.4.2 software (http://www.tree.bio.ed.ac.uk/). The presence of a signal peptide in THSAbf was checked using the SignalP4.1 server (http://www.cbs.dtu.dk/services/SignalP/), entering UNIPROT sequence B1A0T7 in Fasta format as the query.

For protein modeling, a structural model of THSAbf (GENBANK ABZ10760) was generated by homology modeling using M4T, which selects and optimally combines multiple template structures benefitting from the unique contribution of each template [25]. Selected templates were the GH51 α-l-arabinofuranosidases from *Geobacillus stearothermophilus* [8] (PDB code 1qw9) and *Thermobacillus xylanilyticus* [4] (PDB code 2vrq). The quality and stereochemistry of the models were assessed using ProSA-II (http://www.prosa.services.came.sbg.ac.at/prosa.php) and PROCHECK (http://www.ebi.ac.uk/thornton-srv/software/PROCHECK/), respectively. Prior to ligand docking, an array of representative protein conformations defining the conformational space of THSAbf was generated using CONCOORD (http://www3.mpibpc.mpg.de/groups/de_groot/concoord/) and GROMACS (http://www.gromacs.org/) as follows. Using the structural model of THSAbf as the starting conformation, geometrical constraints were defined using the van der Waals parameters from the OPLS-AA force field and CONCOORD’s default parameters for atomic bonds and angles. However, chirality was assessed ‘on the fly’, and the ‘bump check’ option was activated to avoid steric clashes. A total of 500 conformations were generated using the DISCO application in CONCOORD, adjusting the number of trials per run and iterations per structure to 100 and 4000, respectively. In the final stage, structural clustering of the conformers was performed using the *g_cluster* application in GROMACS implementing the *gromos* clustering algorithm and a root mean square deviation cutoff of 2Å. This resulted in 25 clusters, whose centroids were used as representative conformations. The final structural models of the different substrates-bound THSAbf were derived using AutoDock Vina (ADV1.1.2, http://www.vina.scripps.edu/) as follows. The structures of d-xylose, xylobiose and xylotriose, and the branched pentasaccharide XA^3^XX and xylopentaose were obtained from X-ray structures deposited in the PDB databank (1px8, 1gor, 1b30, 2vrq and 3wn2). To adapt these to the requirements of ADV, polar hydrogen atoms and Gasteiger–Marsili charges were added to the ligands using AutoDock tools implemented in MGL Tools version 1.5.6 (http://www.mgltools.scripps.edu), before docking them onto all modeled conformers of THSAbf using a docking grid box centered on the two catalytic glutamic acid residues (E177, and E296) with a size of 45 × 40 × 60 Å^3^. The docking poses were ranked by their predicted binding energy (as per ADV implementation) and the top ten poses were considered for each pair THSAbf–ligand representative conformers. Finally, poses were visually inspected and selected based on the correct and putative productive geometry between catalytic residues and substrates.

### Enzyme activity measurements

Routinely, activity measurements were performed in 50 mM sodium phosphate buffer, pH 6.5, containing BSA (1 mg/mL) and *p*NP-Ara*f* (4 mM final concentration). Before adding THSAbf, the mixture was pre-incubated for 5 min at 60 °C in a water bath, and then 100 µL of THSAbf solution was added and incubation pursued. The total volume of the reaction mix was 1 mL. Aliquots (100 µL) of the reaction mixture were removed at regular intervals over a 14-min incubation period and immediately added to 500 µL of cold (0 °C) 1 M Na_2_CO_3_. To read absorbance values (at 401 nm), a 200 µL aliquot of the mixture was transferred to a microtiter plate that was then placed in a Sunrise UV/Vis microspectrophotometer (Tecan Ltd, Männedorf, Switzerland). Reaction rates, measured in terms of µmoles released per min, were determined by comparing absorbance values with a standard curve prepared using known concentrations of *p*NP-OH (over the range 0 to 1 mM) and 1 U of activity was defined as the quantity of THSAbf required to release 1 µmol *p*NP-OH per min.

To determine the effect of pH on THSAbf activity, the above method was performed at 37 °C using *p*NP-Ara*f* and *p*NP-Xyl*p* as substrates, or the DNS method was employed to measure the release of reducing sugar from LVWAX. Reactions were performed at different pH values using either sodium citrate (pH 3–6), sodium phosphate (pH 6–8), or citrate–phosphate (pH 5.5–6.5) buffer to cover the whole range from pH 3 to 8. Four independent measurements were performed for each pH value and standard deviations were calculated. Similarly, to determine the effect of different temperatures on activity, measurements were performed as described, but covering a range of temperatures, from 30 to 85 °C, collecting four independent measurements for each temperature and calculating standard deviations. Regarding enzyme thermostability, this was determined by incubating samples of THSAbf in 50 mM sodium phosphate, pH 6.5, containing BSA (1 mg/mL) at different temperatures (50, 60, 65, 70 °C). Aliquots (100 µL) were removed at regular intervals and activity was assayed as described above (at 60 °C). Thermostability measurements were performed in duplicate.

### Determination of kinetic parameters

Fourteen different substrates, including *p*NP-α-l-arabinofuranoside, pNP-Ara*f*; *p*NP-β-d-xylopyranoside (*p*NP-Xyl*p),p*NP-β-d-galactofuranoside (pNP-Gal*f*), *p*NP-β-d-galactopyranoside, *p*NP-β-d-glucopyranoside, *p*NP-α-l-glucopyranoside, *p*NP-β-d-fucopyranoside, *p*NP-α-l-fucopyranoside, *p*NP-β-l-fucopyranoside*, p*NP-α-d-mannopyranoside, *p*NP-β-d-mannopyranoside, *p*NP-α-l-arabinopyranoside, *p*NP-β-l-arabinopyranoside, and *p*NP-α-l-rhamnopyranoside, were used to perform the initial tests. Based on the results, the Michaelis–Menten parameters, *k*_cat_ and *K*_M_, were determined for the hydrolysis of three different *p*NP-Ara*f*, *p*NP-Xyl*p* and *p*NP-Gal*f* by THSAbf. All reactions were performed in triplicate at 60 °C in 50 mM sodium phosphate, pH 6.5, containing BSA (1 mg/mL). To investigate the relationship between substrate concentration, [S] and reaction velocity, ν, the following substrate concentrations were employed: *p*NP-Ara*f*, 0–5 mM; *p*NP-Xyl*p,* 0–40 mM; *p*NP-Gal*f*, 0–14 mM. Afterward, data were analyzed using the enzyme kinetics module of Sigma Plot 10.0 software and the Michaelis–Menten model.

### Assessing the activity of THSAbf on complex substrates

The activity of THSAbf was measured on several polymeric substrates. These included branched sugar beet and linear arabinans (Megazyme), and xylans from birch and beechwood. Reactions contained the substrate (10 mg/mL) dissolved, or partially suspended, in 900 µL of 50 mM sodium phosphate, pH 6.5, containing BSA (1 mg/mL). THSAbf-mediated hydrolysis was performed at 60 °C and the time-dependant progress of the reaction was assessed in a discontinuous assay, in which aliquots (100 µL) were removed at regular intervals to measure the quantity of soluble reducing sugars using the DNS method [26]. To account for different substrate specificities, the number of units (defined based on the release of *p*NP-OH from *p*NP-Ara*f*) of THSAbf used in the reactions was different (0.85 and 1.7 U for birch and beechwood xylans, respectively, and 400 and 1200 U for the branched and linear arabinans, respectively). Results, obtained by measuring absorbance at 540 nm, were compared to standard curves prepared using either d-xylose or l-arabinose (solubilized in reaction buffer containing complex substrate at the initial concentration). One unit of THSAbf activity was defined as the quantity of enzyme required to release 1 µmol arabinose or xylose equivalent per minute. To determine the kinetic parameters, *k*_cat_ and *K*_M(app)_, for THSAbf-catalyzed hydrolysis of arabinans, substrate concentrations were varied, either in the range 10–140 mg/mL (sugar beet arabinan) or in the range 2–19 mg/mL (linear debranched arabinan). The low solubility of linear arabinan required the use of a Thermomixer Comfort (Eppendorf, Hamburg, Germany) operating at 60 °C and 1500 rpm.

### Hydrolysis of wheat arabinoxylan and bran using hemicellulolytic cocktails

First, to study the action of THSAbf and other enzymes on low viscosity wheat arabinoxylan, or LWAX (Megazyme) and destarched wheat bran, or DWB (ARD, Pomacle, France), the monosaccharide composition of these substrates was determined using acid hydrolysis, followed by separation and quantification of the monosaccharides by HPAEC-PAD, using a DIONEX system (Fisher Scientific, Strasburg, France) equipped with a Carbopac SA10 column and pre-column equilibrated in an aqueous solution of 1 mM sodium hydroxide. Analyses were performed in triplicate (*n* = 3), over a 30-min period, using a flow rate of 1.2 mL min^−1^ and an eluent composed of an aqueous solution of 100 mM sodium hydroxide. Quantitative data analysis was performed referring to linear concentration–response reference curves prepared using the same column and five different monosaccharide solutions in the range 0 to 5 mg L^−1^ (Table 1).

**Table 1:** A. Monosaccharide composition of LWAX and B. monosaccharide composition of DWB

	Quantity mmol/g	% wt	Xyl/Ara ratio
A. Destarched wheat bran			
Xylose	1.95 ± 0.2	47.21	1.74
Arabinose	1.12 ± 0.22	27.04	
Glucose	0.07 ± 0.01	1.79	
Galactose	0.96 ± 0.16	23.33	
Mannose	0.03 ± 0.01	0.64	
B. Low-viscosity wheat arabinoxylan			
Xylose	2.89 ± 0.17	57.28	1.31
Arabinose	2.20 ± 0.10	42.02	
Glucose	0.02	0.32	
Galactose	0.02	0.38	
Mannose	ND	0	

*n* = 3 and SD values are shown

Afterward, the action of THSAbf on LVWAX and DWB was compared with that of the GH51 Abf and the GH11 β-d-xylanase from *Thermobacillus xylanilyticus* (*Tx*Abf and *Tx*Xyn, respectively) and the possible synergy between THSAbf and these enzymes was investigated. To achieve this, eight reactions were set up for each substrate, mixing a substrate suspension (in 50 mM sodium phosphate, pH 6.5, containing 1 mg/mL BSA and pre-incubated at 60 °C) with the following: (1) water (control), (2) THSAbf, (3) *Tx*Xyn, (4) *Tx*Abf, (5) THSAbf + *Tx*Xyn, (6) THSAbf + *Tx*Abf, (7) *Tx*Abf + *Tx*Xyn and finally (8) THSAbf + *Tx*Xyn + *Tx*Abf. Regarding enzyme concentrations, THS and *Tx*Abfs were used at 15 U/mL (activity determined on *p*NP-Araf), while *Tx*Xyn was used at 6.25 U/mL (activity determined on birchwood xylan). To conduct the reactions, substrates (20 mg) were thoroughly rehydrated in sodium phosphate, pH 6.5, for 16 h at 60 °C under stirring, then the enzyme was added. Aliquots were removed at several intervals during a 48 h period and the supernatants were immediately isolated by centrifugation at 20,000×*g* and 4 °C for 5 min, and then stored frozen (−20 °C) while awaiting analysis. To identify and quantify the reaction products, samples were injected onto a Carbopac PA100 column running on an HPAEC-PAD system (Dionex). Briefly, having equilibrated the column in 150 mM sodium hydroxide containing sodium acetate (5 mM), elution of the different reaction products was achieved using a gradient of increasing sodium acetate concentration (5–85 mM over 30 min, then 500 mM for 3 min, followed by a return to 5 mM for 17 min). To calibrate the analysis, internal standards (d-xylose, l-arabinose, xylobiose, xylotriose, xylotetraose, xylopentaose, xylohexaose) were injected at concentrations ranging from 0 to 25 mg L^−1^. Results were analyzed using Chromeleon 6.8 software to perform peak integration of the chromatogram.

## Results and discussion

### Strain isolation, gene discovery, cloning, and analysis

A microbial collection campaign performed in Tunisia in 2004 allowed the isolation of a bacterial species from a soil sample (collected in the region of Gafsa) on the basis of its ability to grow at 55 °C in the culture medium. Sequencing of the rDNA of the bacterial isolate and comparison with entries in the SILVA ribosomal database revealed that it was closely related to several *Paenibacillus* strains, with *Paenibacillus* sp. MK17 (EF173331) being its nearest neighbor. Accordingly, the isolate was designated *Paenibacillus* sp. *HanTHS1*.

The creation and subsequent screening of a clone library constructed using *Paenibacillus* sp. *HanTHS1* genomic DNA revealed seven individual isolates that formed a blue color at the colony level in the presence of X-Xyl. Plasmid extraction from one of these and DNA sequence analysis revealed the presence of a DNA insert composed of 4464 bp. Further analysis disclosed three ORFs, one of which (1521 bp) was identified by BLASTX (http://www.blast.ncbi.nlm.nih.gov/Blast.cgi) as a family 51 glycoside hydrolase (http://www.cazy.org/GH51). The sequence of this ORF, designated *abf*A, was deposited in GENBANK (accession number ABZ10760). Further analysis of the BLASTX results revealed that the amino acid sequence encoded by *abf*A is 100 % identical to that of the Abf (GH51) from *Paenibacillus* sp. oral taxon 786 str D14 (UniProt C6IZP3) and displays >80 % homology with GH51 Abfs from *Paenibacillus lactis* 154 (G4HDR3), *Paenibacillus* sp. HGF5 (F3M674), *Paenibacillu*s sp. Y412MC10 (D3ELR9), and *Paenibacillus* vortex V453 (E5YTZ2), results that further support the conclusion that the Tunisian bacterial isolate belongs to the genus *Paenibacillus*. Moreover, analysis of the translated *abf*A sequence using the SignalP 4.1 server failed to reveal the presence of an N-terminal signal peptide, indicating that the native protein is possibly located in the cytoplasm.

Phylogenetic analysis of non-redundant sequences in family GH51, including that of THSAbf, revealed an unrooted tree in which Abfs of fungal, plant and viral origin are clustered into three relatively well-defined regions. Moreover, the GH51 members that are annotated in the CAZy database as cellulases (EC 3.2.1.4) are localized in a part of the tree that is very distinct from that harboring THSAbf (Additional file 1). Indeed, the latter forms part of a different subgroup that contains the Abf from *Paenibacillu*s sp. Y412MC10 (D3ELR9) and the sequence of the well-characterized *Gs*Abf (B3EYN4).

Cloning of *abf*A in pET21 under the control of the T7 promoter facilitated the expression of recombinant THSAbf in *E. coli*. Unusually, THSAbf could be expressed without induction by IPTG, even though no lactose or other analog was present in the culture medium. This advantageous feature was not further investigated, but it is possible that auto-induction is of the leakiness of the Lac-UV5 lac promoter. Working in this way, the average final pure protein yields were in the range of 20–25 mg L^−1^ culture, whereas IPTG-induced protein production led to approximately twofold lower yields.

### Activity of THSAbf and E296A on aryl-monosaccharides and polymeric substrates

Rapid screening of THSAbf activity, using 14 different aryl-monosaccharides, representing both furanose and pyranose conformations and sugars from d- and l-series, confirmed that THSAbf is specific for sugars displaying related conformations, notably *p*NP-Ara*f*, *p*NP-Gal*f* and *p*NPXyl*p*. THSAbf was most active on *p*NP-Ara*f*, with its specific activity on this substrate being 27- and 2300-fold higher than that on *p*NP-Gal*f* and *p*NP-Xyl*p*, respectively (Table 2). These results reveal that like the majority of GH51 glycoside hydrolases [27], THSAbf can best be described as an Abf and that the subsite −1 has a clear preference for the furanose conformation. Further testing on polymeric substrates revealed that, like the recently described Abfs from *Cellulomonas fimi* ATCC 484 and *Alicyclobacillus* sp. A4 [18, 28], THSAbf could release reducing sugars from a variety of polysaccharides, including branched and linear sugar beet arabinans (SA of 1.1 ± 0.24 and 1.8 ± 0.1 U mg^−1^, respectively), and xylans from birch and beechwood (SA of 10.8 ± 0.1 and 14.5 ± 1.3 U mg^−1^ respectively). While it was unsurprising to detect activities on sugar beet arabinan (*k*_cat_/*K*_M_ of 0.194 s^−1^ mg^−1^ mL), which bears l-arabinosyl ramifications, it was more surprising to detect activity on linear debranched arabinan (*k*_cat_/*K*_M_ of 0.196 s^−1^ mg^−1^ mL), because this suggests that THSAbf can hydrolyze both α-1,3 bonds linking side-chain arabinosyl moieties and α-1,5 bonds linking main-chain arabinosyl moieties. Moreover, it is noteworthy that the detection of activity on 4-*O*-methylglucuronoxylans (i.e., birch and beechwood xylans) was unexpected for a GH51 Abf, since these polysaccharides contain very few α-l-linked arabinosyl moieties. Significantly, unlike wild-type THSAbf, the mutant E296A displayed only very weak (approximately, 1600-fold lower than the wild-type enzyme) on *p*NP-Ara*f* and displayed no measurable activity on *p*NP-Xyl*p* and beechwood xylan, consistent with the fact that the mutant is crippled by the lack of a catalytic nucleophile and that all of the activities attributed herein to THSAbf described are defined by a single active site.

**Table 2:** Kinetics of hydrolysis of aryl-monosaccharides by THSAbf

Substrate	*K*_M_ (mM)	*k*_cat_ (s^−1^)	*k*_cat_/*K*_M_ (s^−1^ mM^−1^)	SA (U mg^−1^)
*p*NP-Ara*f*	0.31 ± 0.1	328 ± 23	1050	426 ± 36
*p*NP-Xyl*p*	23.8 ± 4.1^a^	10.6 ± 0.9	0.44	1.70 ± 5
*p*NP-Gal*f*	8.6 ± 0.9^a^	327 ± 20	38	103 ± 2

^a^These values are subject to caution since the maximum substrate concentration used in each case was less than 2 × *K*_M_. *n* = 3 and SD values are shown

In the light of the above results, it is worth recalling that THSAbf was first revealed by functional screening using a 5-bromo-4-chloro-3-indolyl-β-d-xylopyranoside and that the subsequent discovery of an Abf appeared to be anomalous. However, the fact that THSAbf appears to be multi-functional rationalizes this preliminary finding. The discovery that THSAbf displays an endo-mode of action, hydrolyzing internal β-1,4 bonds between d-xylosyl subunits in heteroxylans and perhaps internal α-1,5 bonds in arabinan is unusual, even if the results also reveal that THSAbf is not a potent endoxylanase. Indeed, when compared with GH11 *Tx*Xyn (approximately, 1500 U mg^−1^ on birchwood), the specific activities of THSAbf on birch and beechwood heteroxylans (10–15 U mg^−1^) are quite low. While this indicates the intrinsic limit of describing THSAbf as an endoxylanase, the results described below indicate that it is rather a question of arabinose substitution levels.

### Influence of pH and temperature on activity

**Fig. 1:**
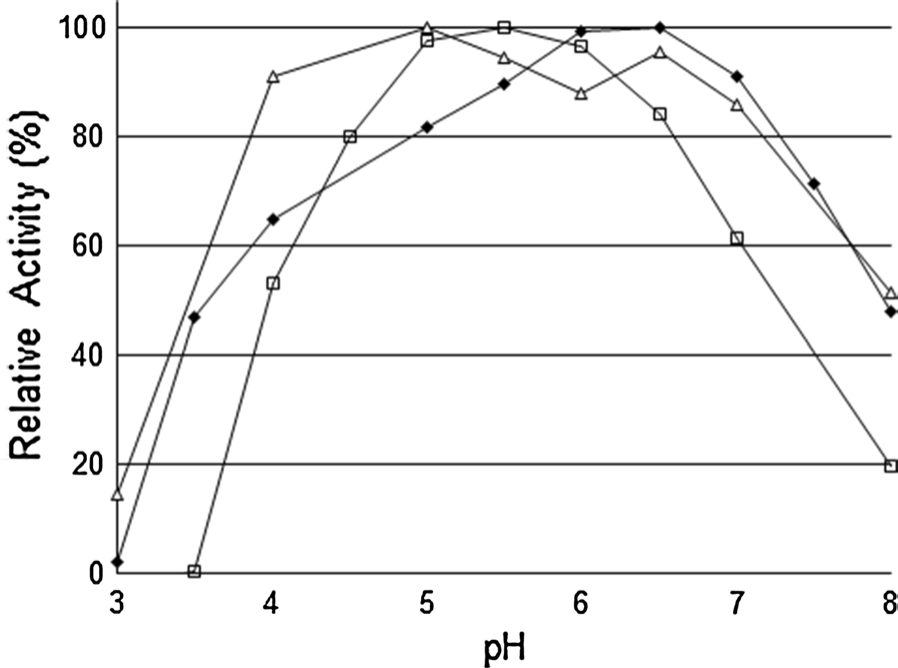
pH-dependent relative activity of THSAbf (*n* = 4). Enzyme activity was measured by monitoring the release of pNP or reducing sugars at different pH values. Various buffer systems were utilized: citrate (pH 3–6), phosphate (pH 6–8), and citrate–phosphate (pH 5.5–6.5). Different substrates were tested. *Filled diamonds* pNP-Ara*f*; *open squares* pNP-Xyl*p*; *open triangles* LWAX. pH profiles for pNP-Ara*f*, pNP- Xyl*p*, and LWAX. 100 % activity was taken as the activity at the optimal pH for a given substrate (relative activity)

The measurement of THSAbf activity on three different substrates (*p*NP-Ara*f*, *p*NP-Xyl*p* and LWAX) at different pH values revealed that activities are near maximal over a relatively wide pH range from approximately 4 to 7, but the actual pH curves are not identical. In particular, the bell-shaped curve representing the pH dependence of the reaction involving *p*NP-Xyl*p* is narrower with maximal activity occurring in the range 5–6, centered on pH 5.5 (Fig. 1), while the curves describing the pH dependence of reactions involving LVWAX and *p*NP-Ara*f* are broader, the latter displaying a rather irregular form. Overall, these results are unexpected and apparently inconsistent with the notion of a single active site in THSAbf. However, it is noteworthy that similar results have been described for other glycoside hydrolases [29, 30]. Moreover, considering that the binding of structurally different substrates within the active site of THSAbf probably induces topological modifications of the active site, it is likely that the distance separating the catalytic residues (nucleophile and acid/base) is altered. This is significant for the optimum pH for activity, because this mainly results from the distance-dependent Coulomb interaction between the catalytic residues and the interaction of these with neighboring residues that participate in an often complex protonic network.

Studying the influence of temperature on THSAbf activity revealed that optimal activity was achieved at 75 °C, although our data also revealed that THSAbf is relatively unstable at this temperature (Additional file 2, A). At 60 °C, the stability of THSAbf was outstanding, since 100 % activity could still be measured after 24 h (Additional file 2, B). Consequently, in subsequent studies, 60 °C was chosen as the preferred operational temperature for THSAbf.

### Activity of THSAbf on isolated wheat arabinoxylan and wheat bran

**Fig. 2:**
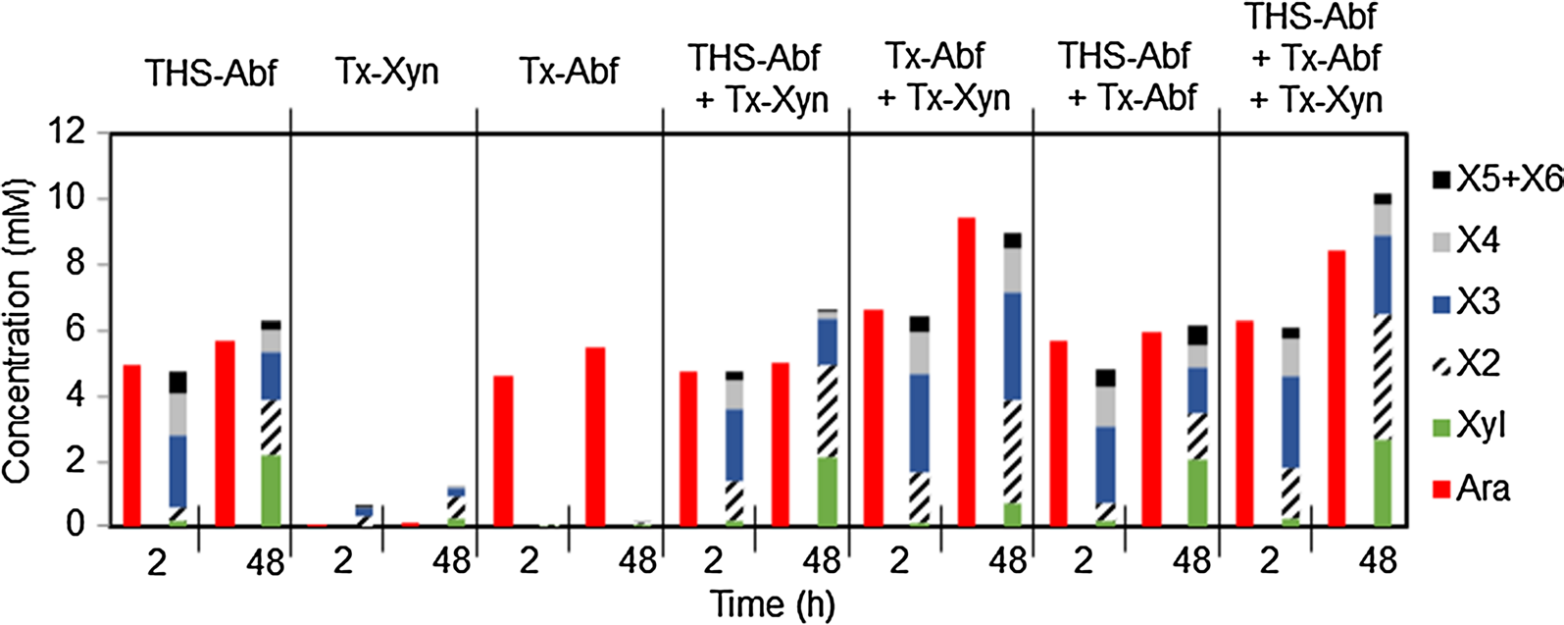
Hydrolysis of LVWAX using single enzymes or enzyme combinations. Histograms were prepared using results from experiments performed in triplicate

Since THSAbf is thermophilic, the activity of this enzyme on complex substrates was studied alone and combined with two other thermostable enzymes *Tx*Abf and *Tx*Xyn. Operating alone, THSAbf, *Tx*Abf and *Tx*Xyn, clearly displayed hydrolytic activity on LVWAX (Fig. 2; Table 3; Additional file 3). Typical of an Abf, *Tx*Abf only released arabinose as the reaction product (25.9 % w/w total arabinose), which is consistent with previous results for this enzyme [11]. Arabinose release by *Tx*Abf was rapid, with almost maximal yields being achieved after only 2 h. Likewise, the hydrolytic behavior of *Tx*Xyn on LVWAX was consistent with the known properties of this enzyme, with xylobiose and xylotriose being the main reaction products, though only 8.9 % w/w total xylose was solubilized. Regarding THSAbf, its behavior on LVWAX was most untypical of a GH51 Abf, because in addition to the presence of soluble arabinose (26.8 % w/w total arabinose), analysis of the reaction products revealed soluble xylose (48.8 % w/w total xylose), which was mainly in the form of xylo-oligosaccharides (xylotriose, xylotetraose and xylobiose in that order). Analysis of the kinetics of product formation revealed that, like arabinose, xylotriose and xylotetraose were formed very rapidly (maximal yields achieved after 1 h), but xylobiose appeared more progressively, possibly being the result of the hydrolysis of higher DP xylo-oligosaccharides.

**Table 3:** Yield of soluble sugars obtained upon incubating LVWAX with different enzymes and enzyme combinations

Enzymes	% w/w yield soluble sugars		
	Arabinose	Xylose (mono)^a^	Xylose (equivalent)^b^
THSAbf	26.8	7.6	48.8
*Tx*Xyn	0.6	1.0	8.9
*Tx*Abf	25.9	0.2	0.6
THSAbf + *Tx*Xyn	23.7	7.5	45.1
*Tx*Abf + *Tx*Xyn	44.6	2.5	85.2
THSAbf + *Tx*Abf	28.0	7.1	51.7
THSAbf + *Tx*Abf + *Tx*Xyn	39.8	9.3	79.9
Control (no enzyme)	0.2	0.0	0.1

^a^Soluble monosaccharide

^b^Total soluble xylose; *n* = 3

Combining the action of the enzymes (THSAbf, *Tx*Abf and *Tx*Xyn), either pairwise or all together, revealed some logical catalytic interplay on LVWAX (Fig. 2; Additional file 4). For example, when *Tx*Abf was deployed simultaneously with *Tx*Xyn, the yields of soluble arabinose and xylose were increased (44.6 % w/w total arabinose and 85.2 % w/w total xylose), with the quantity of soluble xylose equivalents being almost tenfold higher (Fig. 2; Table 3). In sharp contrast, when THSAbf was used with *Tx*Xyn, no benefits were evidenced, since the yields of soluble products were almost identical to those obtained using THSAbf alone. Similarly, using the two Abfs (THSAbf and *Tx*Abf) together yielded results similar to those obtained with THSAbf alone, although the overall yield of soluble arabinose and xylose equivalents was slightly higher. Finally, using all three enzymes simultaneously, quite high solubilization yields were procured (39.8 % w/w total arabinose and 79.9 % w/w total xylose), although these values were lower than those obtained using *Tx*Abf and *Tx*Xyn together. Nevertheless, a significant increase in the yield of soluble monomeric xylose (9.3 % w/w total xylose) could be clearly attributed to the presence of THSAbf.

**Fig. 3:**
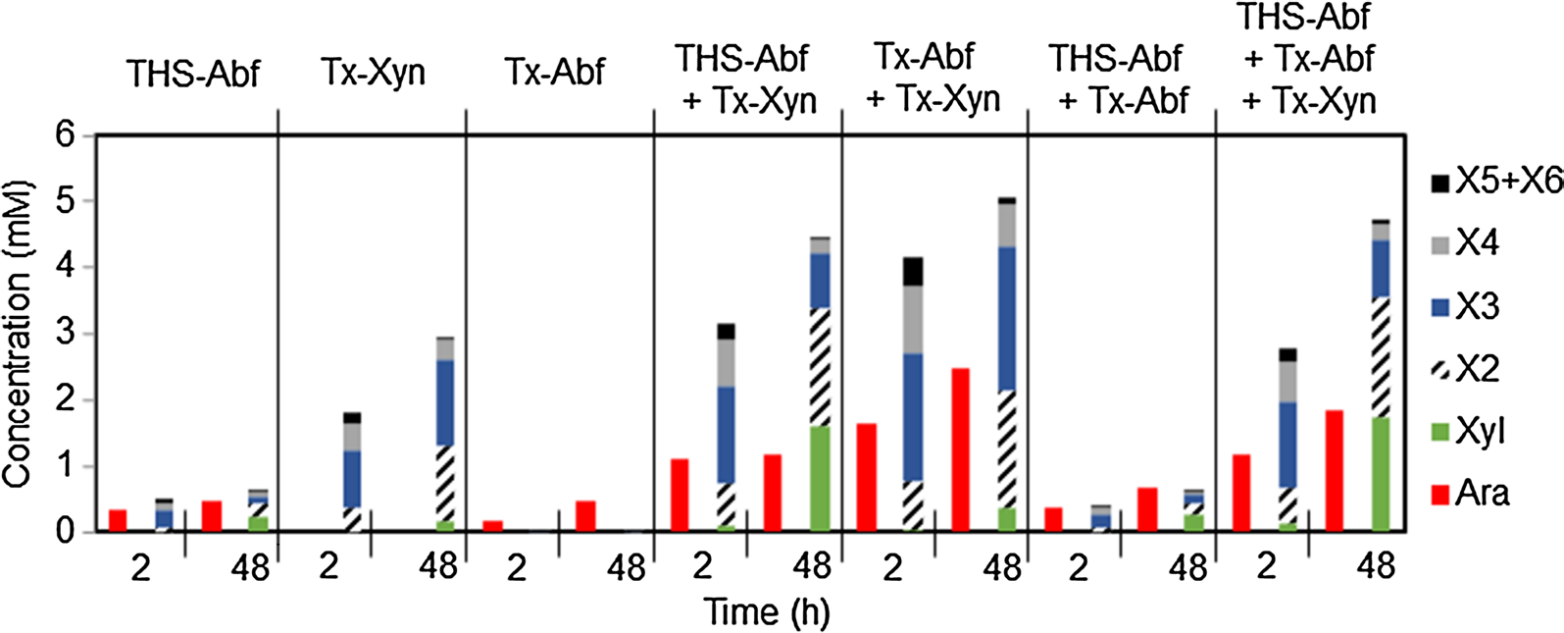
Hydrolysis of DWB using single enzymes or enzyme combinations. Histograms were prepared using results from experiments performed in triplicate

The treatment of DWB with *Tx*Abf led to the release of arabinose as the sole product, which is consistent with the fact that this enzyme displays typical Abf activity (Fig. 3; Additional file 5). After 48 h, approximately 4.3 % (w/w) of arabinose had been released, slightly higher than previous data [11]. Regarding *Tx*Xyn, its impact on DWB was consistent with previous results [31], since approximately 40 % (w/w) xylose was released, mostly as xylobiose and xylotriose, with a smaller amount of xylose being evidenced. Xylotetraose and xylopentaose were also visible among the products, but logically no free arabinose was formed. Most importantly, the hydrolysis of DWB with THSAbf confirmed the previous results obtained on LVWAX. Compared to *Tx*Abf, although THSAbf solubilized an almost identical amount of arabinose (4.3 % after 48 h), the reaction was much faster (3.2 % solubilized after only 2 h) and was accompanied by the equally fast release of xylotriose and xylotetraose (together representing 5.2 % w/w xylose) and the more progressive solubilization of xylose (1.3 % w/w), thus providing a clear indication that THSAbf possesses the ability to hydrolyze the β-1,4 bonds linking both internal and terminal d-xylosyl moieties.

Combining the action of THSAbf with that of *Tx*Xyn provided overall gains in the solubilization of arabinose and xylose from DWB (Fig. 3; Additional file 6). For example, the quantity of arabinose released into the reaction supernatant was 2.5-fold greater than that obtained using THSAbf alone, and the amount of total xylose released was approximately 18 % higher, with 8.2 % w/w total xylose being released in monosaccharide form (Table 4). Nevertheless, combining *Tx*Abf and *Tx*Xyn procured an even better performance, since 22 % w/w total arabinose and 69.6 % w/w total xylose were released in soluble form, although the amount of soluble xylose in monosaccharide form represented a little less than 2 % w/w total xylose. Predictably, the association of the two Abfs, THSAbf and *Tx*Abf, for the treatment of DWB yielded a composite product profile, with approximately 6 % w/w total arabinose and 7 % w/w total xylose being recovered in soluble form, the latter represented by xylo-oligosaccharides and a small amount of xylose (1.5 % w/w total xylose). Finally, the simultaneous deployment of all three enzymes did not procure added value when compared with the deployment of *Tx*Abf and *Tx*Xyn. Indeed, the overall yields of soluble xylose and arabinose were lower (approximately, 47 % w/w total xylose and 16.5 % w/w total arabinose, respectively) when compared with the dual action of *Tx*Abf and *Tx*Xyn (which solubilized approximately 69 % w/w total xylose and 22 % w/w total arabinose), though the soluble monosaccharide (xylose) production was significantly higher (8.93 % w/w, instead of 1.9 % w/w total xylose).

**Table 4:** Yield of soluble sugars obtained incubating DWB with different enzymes and enzyme combinations

Enzymes	% w/w yield soluble sugars		
	Arabinose	Xylose (mono)^a^	Xylose (equivalent)^b^
THSAbf	4.29	1.32	6.47
*Tx*Xyn	0.00	0.98	39.68
*Tx*Abf	4.31	0.04	0.46
THSAbf + *Tx*Xyn	10.74	8.24	44.73
*Tx*Abf + *Tx*Xyn	22.14	1.92	69.58
THSAbf + *Tx*Abf	5.95	1.52	6.94
THSAbf + *Tx*Abf + *Tx*Xyn	16.53	8.93	47.25
Control (no enzyme)	0.18	0.08	0.34

^a^Soluble monosaccharide

^b^Total soluble xylose

Previous work has shown that the heteroxylans in DWB are differentially organized within its component layers. Heteroxylans in the aleurone layer display moderate arabinose substitution (Xyl/Ara ratio of 2.07), while those in the middle and outer layers of DWB are characterized by lower Xyl/Ara ratios [32]. *Tx*Xyn very efficiently solubilizes the aleurone arabinoxylans, an observation that correlates well with this enzyme’s preference for heteroxylans displaying lower levels of arabinose substitution [31]. Inversely, taking into account the poor activity of THSAbf on birch and beechwood heteroxylans, presumably the activity of THSAbf on the aleurone layer arabinoxylans is also lower than that of *Tx*Xyn. A further indication of this clear distinction between endoxylanase activities of THSAbf and *Tx*Xyn is provided by the comparison of their action on LVWAX. On this substrate, THSAbf is clearly more efficient than *Tx*Xyn, which correlates well with the higher level of arabinose substitution (Xyl/Ara ratio of 1.3). Presumably, on LVWAX THSAbf benefits from the synergy between its arabinofuranosidase and endoxylanase activities. When considering the potential usefulness of THSAbf in xylanolytic cocktails, it is interesting to note that its interplay with other enzymes was not intuitive, in the sense that THSAbf appeared to compete with, rather than complement, the action of a GH11 xylanase. This observation contrasts somewhat with the fact that *Al*Abf51 was reported to complement the action of a GH10 xylanase. However, unlike GH10 xylanases, those from GH11 display rather narrow active site clefts (average 5.5 Å) [33] that preclude the binding of decorated d-xylosyl moieties in either subsite −1 or +1, and only l-arabinosyl (and not other substitutions) is generally tolerated in subsite −2. Therefore, the results presented in this study might imply that THSAbf and *Tx*Xyn vie for the undecorated regions of heteroxylans. In this respect, it is noteworthy that a similar competition phenomenon was reported between GH10 and GH11 xylanases, which led to a decrease in the release of XOS from wheat arabinoxylan [34]. In the case of THSAbf, primarily an Abf, this is more surprising because one would expect this enzyme to first act on available l-arabinosyl moieties. However, in our experiments, THSAbf was used in 20-fold molar excess when compared with *Tx*Xyn; thus in these conditions it is easy to appreciate why *Tx*Xyn was out-competed.

### Protein modeling and investigation of different THSAbf–ligand complexes

**Fig. 4:**
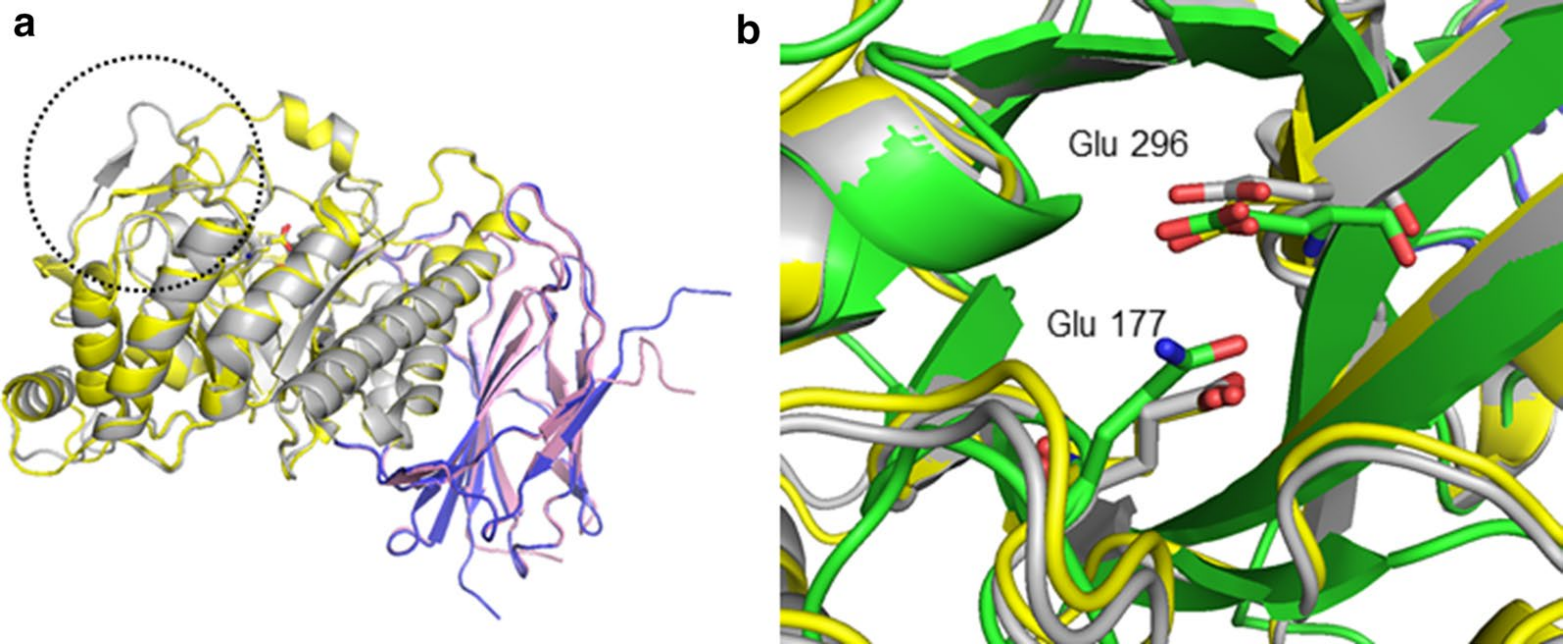
Modeled structures of THSAbf. **a** The overall two domain structure composed of a (β/α)_8_ folded catalytic domain and a C-terminal domain displaying β-sandwich architecture. Two structural conformers of THSAbf (*open gray* and *violet*, *closed yellow* and *pink*) are superposed and the β2α2 loop in two different positions is encircled. **b** Zoom on the catalytic site of the two modeled THSAbf conformers (*gray* and *yellow*) and that of *Gs*Abf (1QW8, *green*). The side chains of the two catalytic residues (Glu 177 and 296 in THSAbf) are shown as sticks. (Figure prepared using PyMOL™ Molecular Graphics System, Version 1.7.2.1)

Attempts to obtain high-quality X-ray diffraction data from THSAbf crystals have so far failed. Therefore, to gain insight into the possible structural determinants of the different activities displayed by THSAbf, structural models were generated and used for ligand docking. Two THSAbf models were built using comparative modeling and templates that display more than 70 % sequence identity with THSAbf, thus ensuring that high-quality structural models were obtained (i.e., Z-score of 6.9 using ProSA-II and absence of major stereo-chemical problems revealed by PROCHECK). Inspection of the overall structure of the two THSAbf models reveals that like all other GH51 Abfs, THSAbf displays two domains, with the catalytic domain displaying (β/α)_8_ architecture and the other a β-sandwich fold (Fig. 4a). Closer analysis of the two THSAbf models reveals that the principal differences between these are loop positions, notably the position of the β2α2 loop. In this respect, it is useful to recall that previous work on *Tx*Abf [4, 35] has shown that the mobility of its β2α2 loop is likely to be an essential feature of catalysis. Nevertheless, when the β2α2 loop of THSAbf is in so-called open configuration, the overall position of the loop is dissimilar to that of *Tx*Abf (2VRQ, chains C); residue Trp101 (borne on the β2α2 loop) is distant from the predicted subsite −1 and the overall active site topology of THSAbf resembles that of the wide *Gs*Abf active site (Additional file 7). On the other hand, in the closed form, the β2α2 loop of THSAbf adopts a configuration that is similar to that of the so-called closed form of *Tx*Abf (2VRQ, chains A and B), with Trp101 superposing well with its equivalent (Trp99) in *Tx*Abf (Additional file 8). Nevertheless, the active site topology remains wider than that of *Tx*Abf, because of the presence of a shortened β6α6 loop. Moreover, like *Gs*Abf, THSAbf displays a long β7α7 loop that is positioned in much the same way as its analog in *Gs*Abf. Likewise, superposition of the structures of *Gs*Abf and *Tx*Abf on the models of THSAbf reveals that the spatial position of the catalytic dyad (E177 and E296) coincides well with those of *Gs*Abf (E175 and E294 in 1QW8,) and *Tx*Abf (E176 and E298, not shown), and is localized just above a clearly defined cavity, which presumably constitutes the subsite −1 (Fig. 4b).

**Fig. 5:**
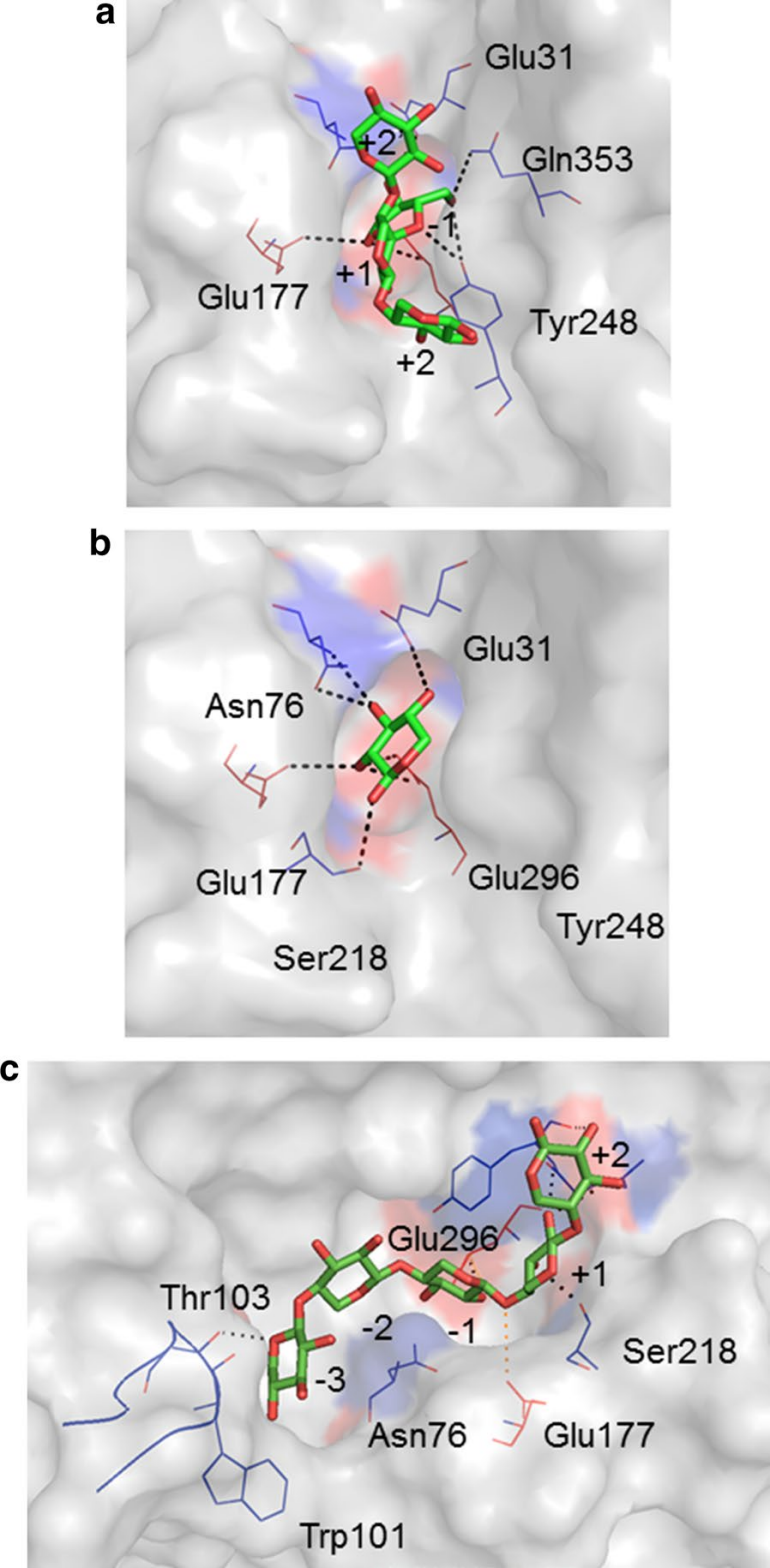
Docking of various ligands into the active site of THSAbf. **a** XA^3^XX, from 2VRQ, **b** a single β-d-xylosyl moiety from 1QW8 and **c** xylopentaose (X5). Sugar moieties in the oligosaccharides are numbered according to their position with regard to the putative scissile bond, with most negative number designating the non-reducing moiety. The side chains of amino acids that might form polar contacts (*dotted lines*) with the sugar ligands are shown (*blue lines*) and the active site residues Glu177 and Glu296 are highlighted using *red lines* and in **c** the β2α2 loop is shown as a *blue ribbon*. (Figure prepared using PyMOL™ Molecular Graphics System, Version 1.7.2.1)

The docking of different putative ligands, including XA^3^XX, a single β-d-xylosyl moiety and xylopentaose (X5) revealed that it was possible to generate satisfactory models of ligand-bound enzymes harboring either an α-l-arabinofuranosyl or a β-d-xylosyl moiety in subsite −1 (Fig. 5). In the case of the oligosaccharide XA^3^XX, the α-l-arabinofuranosyl moiety adopted a position highly similar to that already observed in ligand-bound *Tx*Abf, with the scissile (O4–C1) bond being in a potentially productive position with respect to the two catalytic residues. Importantly, docking revealed that a single β-d-xylosyl moiety could also enter into subsite −1, with its O-5, O-2 and O-3 superposing well with those of a similarly bound α-l-arabinofuranosyl moiety, a result that is consistent with previous findings for *Gs*Abf and with the biochemical data presented here. Even more satisfyingly, ligand docking using X5 provided a rational basis for ligand binding in endo-mode. The combination in the model of THSAbf of a short β6α6 loop and an ‘open’ β2α2 loop creates a rather long and wide active site that can readily accommodate X5. Moreover, the absence of Trp101 in the vicinity of subsite −1 allows the β-d-xylosyl moiety in position 3 to sink quite low into the active, thus properly occupying, subsite −1 and positioning the scissile bond within an almost plausible distance of the catalytic dyad (i.e., 4.1 Ǻ for Glu177-O^ε1^ → O4β-Xyl and 4.3 Ǻ for Glu296-O^ε1^ → C1β-Xyl). Finally, it is noteworthy that the model of the THSAbf:X5 complex predicts that Asn76 Nδ is well positioned to form H-bonds with both Xyl3(O3) and Xyl2(O5), and O^γ1^ of Thr103, which is on the β2α2 loop, is predicted to form a H-bond with Xyl5(O3), the moiety forming the non-reducing end of X5.

Overall, modeling reveals that the position of the β2α2 loop could be a major factor to explain the multi-functionality of THSAbf, especially because a key function has already been attributed to this loop in other GH51 α-l-arabinofuranosidases. Apparently, the mobility of the β2α2 loop could form the basis for the absence of the bulky Trp101 in the active site of THSAbf and thus the ability of the enzyme to productively bind xylooligosaccharides. In this respect, it is perhaps noteworthy that the replacement of Tyr166 by a less bulky amino acid in an unrelated GH43 arabinoxylan hydrolase (AXHd3 from *Humicola insolens*) also provoked a significant modification of active topology and facilitated the productive binding of xylo-oliogosaccharides (DP ≥ 5).

## Conclusion

This work follows the discovery of the bifunctional *Alicyclobacillus* GH51 arabinofuranosidase. Like the former, THSAbf differs from most GH51 Abfs, because it catalyzes the hydrolysis of external and internal glycosidic bonds. However, THSAbf is multifunctional, since it also possesses xylosidase and possibly endo-arabinanase activities. Importantly, the discovery of *Al*Abf51 and THSAbf reveals that current knowledge of glycoside hydrolases is incomplete, especially because only a small fraction of GHs have been characterized and many studies are insufficiently thorough. From an industrial standpoint, we expect that discovery of THSAbf will facilitate the design of simpler, lower-cost xylanolytic cocktails.

## Additional files

10.1186/s13068-016-0550-x Phylogenetic analysis of GH51 sequences. The figure shows an unrooted phylogram built using 577 GH51 sequences. The approximate locations in the phylogram of THSAbf and other characterized GH51 Abfs (*Gs*Abf and *Tx*Abf) are shown.
10.1186/s13068-016-0550-x Thermoactivity and thermostability of THSABF. Figure S2A shows THSAbf activity as a function of temperature and Figure S2B shows thermostability plots.
10.1186/s13068-016-0550-x Hydrolysis of LVWAX by various enzymes. Figure S3A, B and C show the progress of hydrolysis LVWAX by *Tx*Abf(B), *Tx*Xyn and THSAbf respectively. Several reactions products are monitored, including xylose, arabinose and xylooligosaccharides (X2-X6).
10.1186/s13068-016-0550-x Hydrolysis of LVWAX using different enzyme combinations. Figure S4A, B, C and D show the progress of hydrolysis of LVWAX by (A) *Tx*Abf + *Tx*Xyn, (B) THSAbf + *Tx*Xyn, (C) THSAbf + *Tx*Abf and (D) THSAbf + *Tx*Abf + *Tx*Xyn. Several reactions products are monitored, including xylose, arabinose and xylooligosaccharides (X2-X6).
10.1186/s13068-016-0550-x Hydrolysis of DWB by various enzymes. Figure S5A, B and C show the progress of hydrolysis of DWB by *Tx*Abf(B), *Tx*Xyn and THSAbf respectively. Several reactions products are monitored, including xylose, arabinose and xylooligosaccharides (X2-X6).
10.1186/s13068-016-0550-x Hydrolysis of DWB using different enzyme combinations. Figure S6A, B, C and D show the progress of hydrolysis of DWB by (A) *Tx*Abf + *Tx*Xyn, (B) THSAbf + *Tx*Xyn, (C) THSAbf + *Tx*Abf and (D) THSAbf + *Tx*Abf + *Tx*Xyn. Several reactions products are monitored, including xylose, arabinose and xylooligosaccharides (X2-X6).
10.1186/s13068-016-0550-x Modelling alternative active site topologies of THSAbf. Figure S7A and B show proposed models for the so-called open and closed conformers of THSAbf respectively. These are compared to models of active sites (Figure S7C) *Gs*Abf (1QW8 chain B) and (Figure S7D) *Tx*Abf (2VRQ chain C).
10.1186/s13068-016-0550-x Comparison of the position of the β2α2 loop in the closed and open forms of THSAbf and *Tx*Abf. Figure S8A and B show two views (rotation of 90° through X-axis) of the active site with a zoom on the β2α2 loop.

## Abbreviations

Abf: α-l-arabinofuranosidase
Ara: arabinose
DWB: destarched wheat bran
GH: glycoside hydrolase
HPAEC-PAD: high-performance anion-exchange chromatography with pulsed amperometric detection
LB: Luria Bertoni
LVWAX: low-viscosity wheat arabinoxylan
pNP: 4-nitrophenol
pNP-α-l-arabinofuranoside: pNP-Araf
pNP-β-d-galactofuranoside: pNP-Galf
pNP-β-d-xylopyranoside: pNP-Xylp
SA: specific activity
XOS: xylooligosaccharides
Xyl: xylose
X-Xyl: 5-bromo-4-chloro-3-indolyl-β-d-xylopyranoside
