# Supplementary material for: The GH51 α-l-arabinofuranosidase from Paenibacillus sp. THS1 is multifunctional, hydrolyzing main-chain and side-chain glycosidic bonds in heteroxylans
Source: Biotechnol Biofuels. 2016 Jul 8;9:140. doi: 10.1186/s13068-016-0550-x (PMC4939007; doi:10.1186/s13068-016-0550-x)

Figure S1 - Phylogenetic analysis of GH51 sequences. The unrooted phylogram was generated using Figtree 1.4.2 software and data from a bioinformatics pipeline. In total 577 sequences are represented out of an original batch of 984 non-redundant GH51-encoding sequences. The different colors highlight major groups and the letters indicate positions of single sequences or subgroups. A, identifies the approximate position in the phylogram of THSAbf and *Gs*Abf, while B indicates the position of *Tx*Abf. C denotes the subgroup of plant-derived GH51 Abfs and D denotes those of fungal origin. E and G pinpoint parts of the phylogram that harbor sequences that have been annotated with the EC number 3.2.1.4 and F indicates sequences of viral origin.


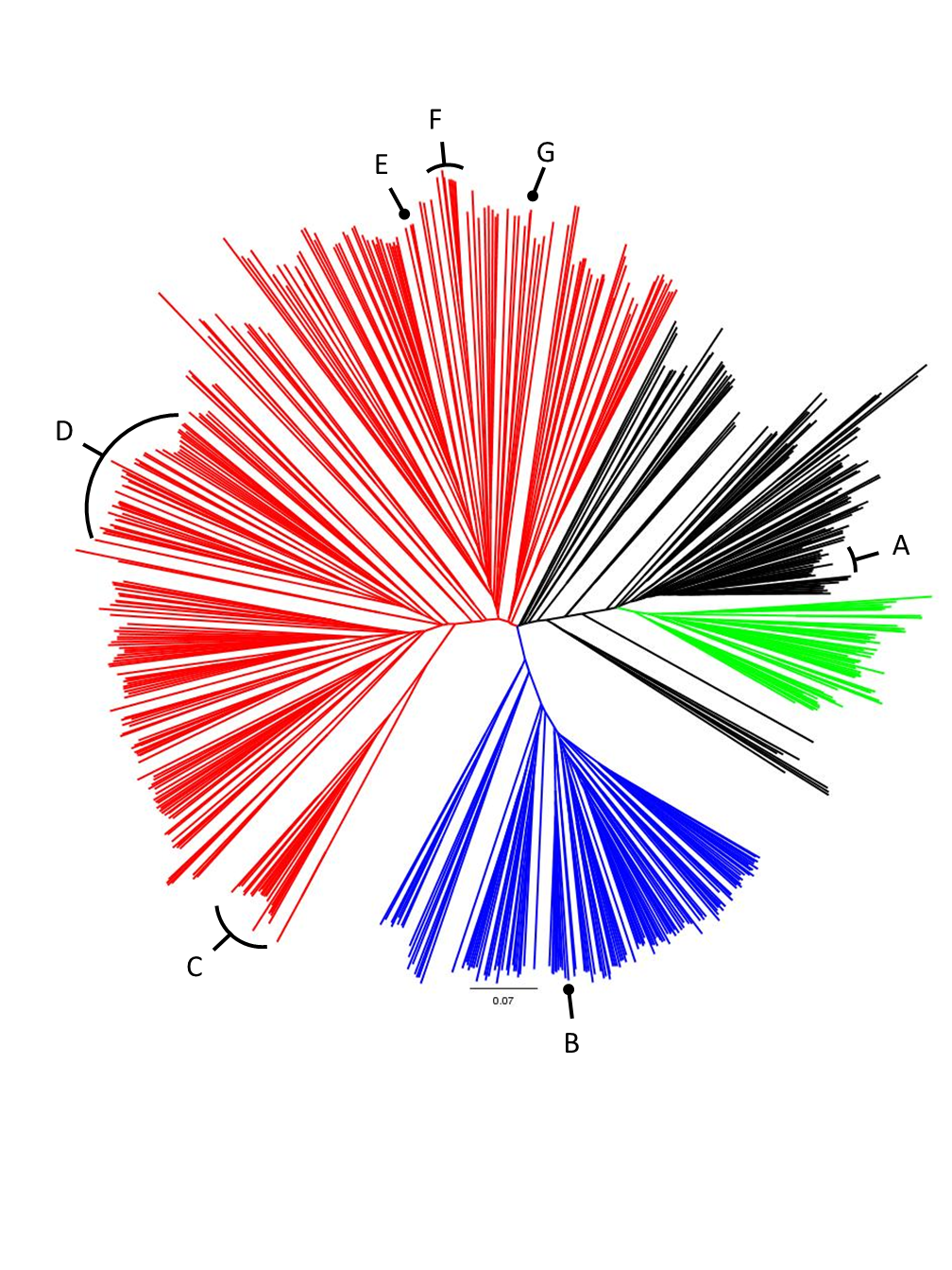

Supplement: Supplementary file 1 — 10.1186/s13068-016-0550-x Phylogenetic analysis of GH51 sequences. The figure shows an unrooted phylogram built using 577 GH51 sequences. The approximate locations in the phylogram of THSAbf and other characterized GH51 Abfs (GsAbf and TxAbf) are shown. [file 13068_2016_550_MOESM1_ESM.docx]
