# Supplementary material for: The GH51 α-l-arabinofuranosidase from Paenibacillus sp. THS1 is multifunctional, hydrolyzing main-chain and side-chain glycosidic bonds in heteroxylans
Source: Biotechnol Biofuels. 2016 Jul 8;9:140. doi: 10.1186/s13068-016-0550-x (PMC4939007; doi:10.1186/s13068-016-0550-x)
Supplement: Supplementary file 2 — 10.1186/s13068-016-0550-x Thermoactivity and thermostability of THSABF. Figure S2A shows THSAbf activity as a function of temperature and Figure S2B shows thermostability plots. [file 13068_2016_550_MOESM2_ESM.docx]

Figure S2 – Thermoactivity (A) and thermostability (B) of THSABF. Thermoactivity experiments were performed in quadruplicates using *p*NP-Araf as substrate. Themostability at each temperature (55°C, ⯁; 60°C, ■; 75°C, ▲) were performed in duplicate.

Fig. 2

A.


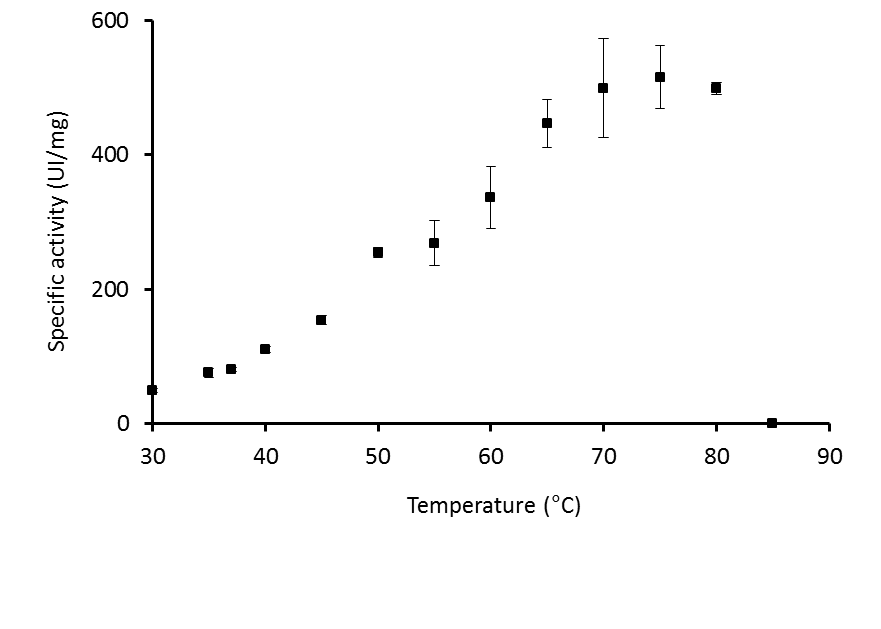


B.
