# Supplementary material for: The GH51 α-l-arabinofuranosidase from Paenibacillus sp. THS1 is multifunctional, hydrolyzing main-chain and side-chain glycosidic bonds in heteroxylans
Source: Biotechnol Biofuels. 2016 Jul 8;9:140. doi: 10.1186/s13068-016-0550-x (PMC4939007; doi:10.1186/s13068-016-0550-x)

**Additional file 3**

Hydrolysis of LVWAX by (A) THSAbf, (B) *Tx*Xyn and (C) *Tx*Abf. The symbols used are arabinose, ◇; xylose, ■; xylobiose, ▲; xylotriose, ○; xylotetraose, △; xylopentaose, ●; and xylohexaose, 🞩. (n=3)

A.


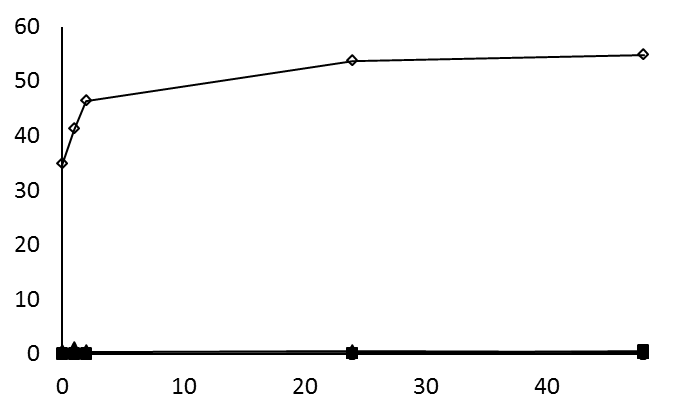


B.


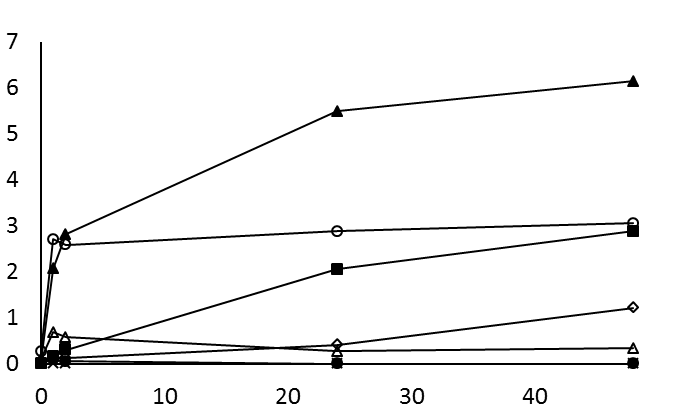


C.


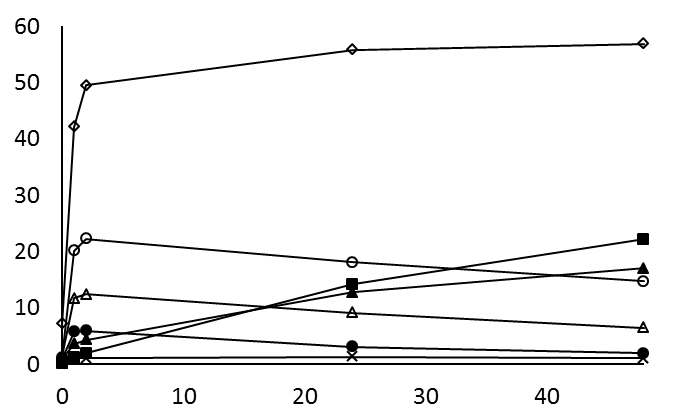

Supplement: Supplementary file 3 — 10.1186/s13068-016-0550-x Hydrolysis of LVWAX by various enzymes. Figure S3A, B and C show the progress of hydrolysis LVWAX by TxAbf(B), TxXyn and THSAbf respectively. Several reactions products are monitored, including xylose, arabinose and xylooligosaccharides (X2-X6). [file 13068_2016_550_MOESM3_ESM.docx]
