# Supplementary material for: The GH51 α-l-arabinofuranosidase from Paenibacillus sp. THS1 is multifunctional, hydrolyzing main-chain and side-chain glycosidic bonds in heteroxylans
Source: Biotechnol Biofuels. 2016 Jul 8;9:140. doi: 10.1186/s13068-016-0550-x (PMC4939007; doi:10.1186/s13068-016-0550-x)

**Additional file 6**

Hydrolysis of DWB using different enzyme combinations combinations (A) *Tx*Abf + *Tx*Xyn, (B) THSAbf + *Tx*Xyn, (C) THSAbf + *Tx*Abf and (d) THSAbf + *Tx*Abf + *Tx*Xyn. The symbols used are arabinose, ◇; xylose, ■; xylobiose, ▲; xylotriose, ○; xylotetraose, △; xylopentaose, ●; and xylohexaose, 🞩. (n=3)

A.


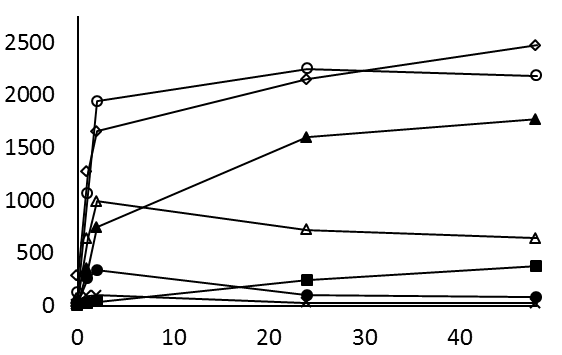


C.


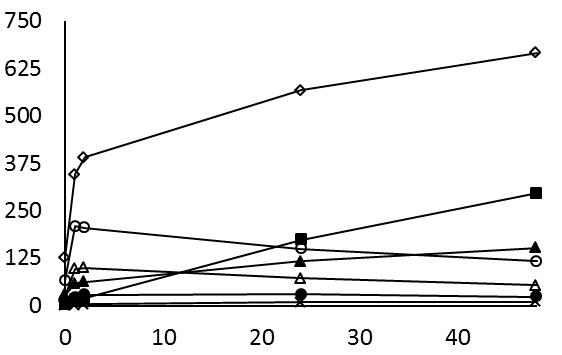


B.


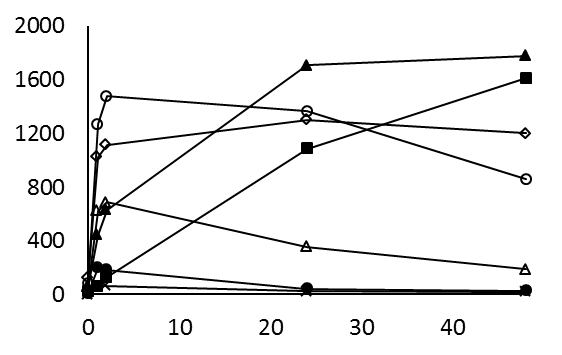


D.


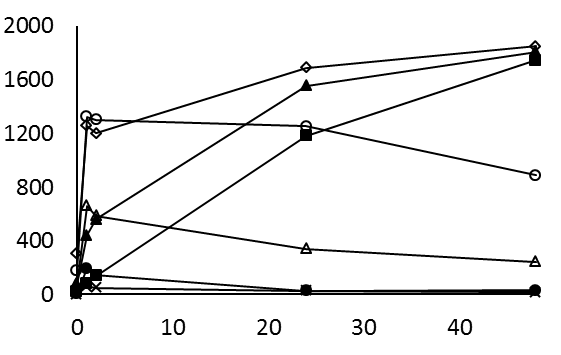

Supplement: Supplementary file 6 — 10.1186/s13068-016-0550-x Hydrolysis of DWB using different enzyme combinations. Figure S6A, B, C and D show the progress of hydrolysis of DWB by (A) TxAbf + TxXyn, (B) THSAbf + TxXyn, (C) THSAbf + TxAbf and (D) THSAbf + TxAbf + TxXyn. Several reactions products are monitored, including xylose, arabinose and xylooligosaccharides (X2-X6). [file 13068_2016_550_MOESM6_ESM.docx]
