# Supplementary material for: The GH51 α-l-arabinofuranosidase from Paenibacillus sp. THS1 is multifunctional, hydrolyzing main-chain and side-chain glycosidic bonds in heteroxylans
Source: Biotechnol Biofuels. 2016 Jul 8;9:140. doi: 10.1186/s13068-016-0550-x (PMC4939007; doi:10.1186/s13068-016-0550-x)

**Additional file 7**

Active site topologies of the A. open and B. closed conformers of THSAbf, compared to the active site topologies of C. *Gs*Abf (1QW8 chain B) and D. *Tx*Abf (2VRQ chain C). In each case the position of the active site nucleophile is highlighted in red. (Figure prepared using PyMOL™ Molecular Graphics System, Version 1.7.2.1).


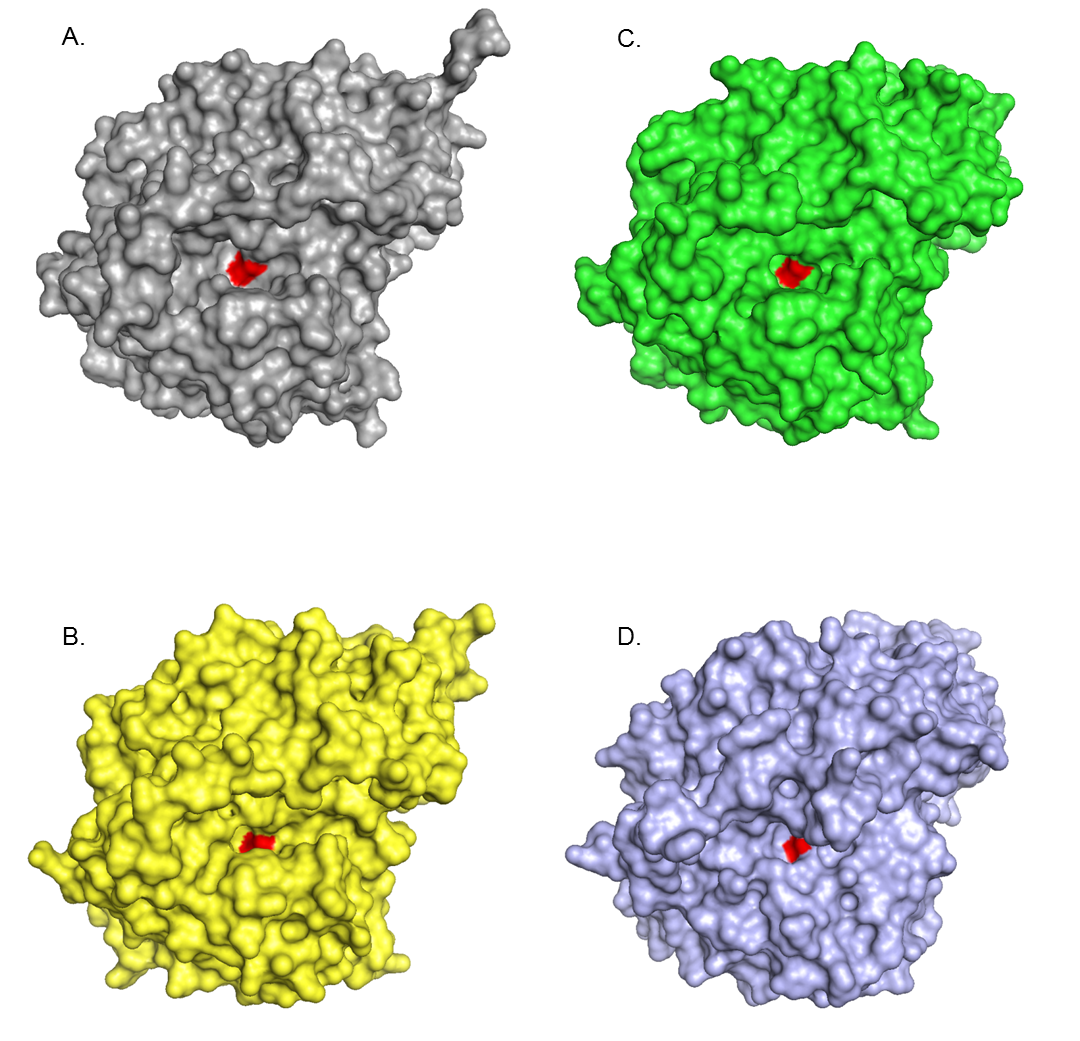

Supplement: Supplementary file 7 — 10.1186/s13068-016-0550-x Modelling alternative active site topologies of THSAbf. Figure S7A and B show proposed models for the so-called open and closed conformers of THSAbf respectively. These are compared to models of active sites (Figure S7C) GsAbf (1QW8 chain B) and (Figure S7D) TxAbf (2VRQ chain C). [file 13068_2016_550_MOESM7_ESM.docx]
