# Supplementary material for: The GH51 α-l-arabinofuranosidase from Paenibacillus sp. THS1 is multifunctional, hydrolyzing main-chain and side-chain glycosidic bonds in heteroxylans
Source: Biotechnol Biofuels. 2016 Jul 8;9:140. doi: 10.1186/s13068-016-0550-x (PMC4939007; doi:10.1186/s13068-016-0550-x)

**Additional file 8**

Two views (rotation of 90° through X-axis) that compare the position of the β2α2 loop in the closed and open forms of THSAbf (yellow and grey respectively) and *Tx*Abf (blue and green respectively). The open form of *Tx*Abf is 2VRQ chain C while the closed form is 2VRQ chain A. In each case the Trp sidechain (101 in THSAbf and 99 in *Tx*Abf) is shown in stick mode. (Figure prepared using PyMOL™ Molecular Graphics System, Version 1.7.2.1).


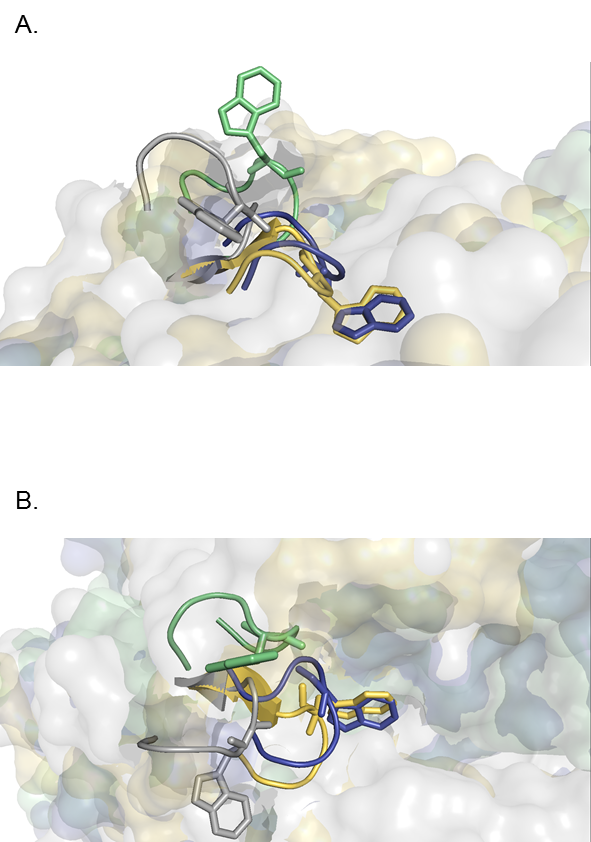

Supplement: Supplementary file 8 — 10.1186/s13068-016-0550-x Comparison of the position of the β2α2 loop in the closed and open forms of THSAbf and TxAbf. Figure S8A and B show two views (rotation of 90° through X-axis) of the active site with a zoom on the β2α2 loop. [file 13068_2016_550_MOESM8_ESM.docx]
